# Transformation of the released asbestos, carbon fibers and carbon nanotubes from composite materials and the changes of their potential health impacts

**DOI:** 10.1186/s12951-017-0248-7

**Published:** 2017-02-20

**Authors:** Jing Wang, Lukas Schlagenhauf, Ari Setyan

**Affiliations:** 1 Institute of Environmental Engineering, ETH Zurich, 8093 Zurich, Switzerland; 2 Advanced Analytical Technologies, Empa, Ueberlandstrasse 129, 8600 Dübendorf, Switzerland

## Abstract

Composite materials with fibrous reinforcement often provide superior mechanical, thermal, electrical and optical properties than the matrix. Asbestos, carbon fibers and carbon nanotubes (CNTs) have been widely used in composites with profound impacts not only on technology and economy but also on human health and environment. A large number of studies have been dedicated to the release of fibrous particles from composites. Here we focus on the transformation of the fibrous fillers after their release, especially the change of the properties essential for the health impacts. Asbestos fibers exist in a large number of products and the end-of-the-life treatment of asbestos-containing materials poses potential risks. Thermal treatment can transform asbestos to non-hazardous phase which provides opportunities of safe disposal of asbestos-containing materials by incineration, but challenges still exist. Carbon fibers with diameters in the range of 5–10 μm are not considered to be respirable, however, during the release process from composites, the carbon fibers may be split along the fiber axis, generating smaller and respirable fibers. CNTs may be exposed on the surface of the composites or released as free standing fibers, which have lengths shorter than the original ones. CNTs have high thermal stability and may be exposed after thermal treatment of the composites and still keep their structural integrity. Due to the transformation of the fibrous fillers during the release process, their toxicity may be significantly different from the virgin fibers, which should be taken into account in the risk assessment of fiber-containing composites.

## Background

A composite material can be defined as a combination of two or more materials that results in better properties than those of the individual components used alone [1]. The composite materials may be preferred because they are stronger, lighter, or less expensive when compared to traditional materials [2]. The components forming the composites can be divided into two main categories: matrix and reinforcement. The continuous phase is the matrix, which can be a polymer, metal, or ceramic [1]. The reinforcement usually adds the strength and stiffness. In most cases, the reinforcement is harder, stronger, and stiffer than the matrix [1]. Fibers with high length-to-diameter ratios are common reinforcement materials. Asbestos fibers were widely used as reinforcement in cement to improve the tensile strength and heat resistance. The most common asbestos-containing industrial material produced worldwide has been cement-asbestos [3]. Carbon fibers with diameters 5–10 μm are used in polymer matrices. With the development of material technology, fibers with smaller diameters are getting popular. Carbon nanotubes (CNTs) with diameters below 100 nm exhibit properties including high strength and tensile stiffness, chirality-dependent electrical conductivity, increased thermal conductivity and one of the highest Young’s modulus [4], therefore they have been considered as a nanofiller for composites.

With the wide applications of fiber-reinforced composites, there come the possibilities of release of the fibers and exposure to workers and consumers. Due to their dimensions, as well as chemical and elemental composition, concerns as to the human health risk associated with exposure to respirable fibers have been vehemently raised [5–7]. The toxicity of fibers is generally determined by the three “D’s”: dose, dimension, and durability [8]. The small aerodynamic diameters of thin fibers enable deposition beyond the ciliated airways. Donaldson et al. [9] provided a schematic with direct comparison between the CNTs and asbestos, showing that the long asbestos and long and stiff CNTs deposit in the parietal pleura and the macrophage cells cannot completely engulf such fibers, resulting in incomplete or frustrated phagocytosis, which leads to oxidative stress and inflammation. In contrast, the short asbestos and compact, entangled CNTs could be cleared by the macrophage cells. The frustrated phagocytosis effect is not limited to CNTs or asbestos, but applicable for high aspect ratio particles [9]. The correlation between biopersistence and adverse pulmonary effects has been demonstrated [10], while the fiber material is of minor importance [11]. Fibers with good biopersistence produce chronic pulmonary inflammation and interstitial fibrosis; if very biopersistent, fibrosis is followed by lung cancer and/or pleural mesothelioma [8]. Defined by the World Health Organization (WHO), respirable fibers have a length above 5 µm, a diameter below 3 µm, and an aspect ratio (length/diameter) above or equal to 3 [12]. The recommended permissible exposure limit (PEL) by the Occupational Safety and Health Administration (OSHA) is 1 respirable fiber/cm^3^ for an 8 h time weighed average [8]. There remains an impending need to undertake research initiatives that focus specifically upon determining the real advantages posed by nanofibers, as well as underpinning their conceivable risk to human health. Both are inextricably linked, and therefore by devising a thorough understanding of the synthesis and production of nanofibers to their potential application and disposal is essential in gaining an insight as to the risk they may pose to human health [6].

The fibers in composite materials can be released to the environment in different phases of the life time of the products, including production and processing, service life, and disposal [13]. Wear and tear, cutting, drilling, sanding, machining, exposure to UV light and heat, chemical erosion, and combustion can all possibly lead to release of fibrous fillers after production. The released fibers may be free standing or partially embedded in the matrix material. Harper et al. [14] suggested that CNT-containing fragments may be turned into household dust. The physical and chemical properties of the fibers can be altered by the mechanical, chemical or thermal energy input during the release process. Therefore, the dimension and biopersistency of the released fibers may be different from the original materials, e.g. asbestos could be entirely transformed to a mixture of non hazardous silicate phases by thermal treatment; large carbon fibers can be turned into respirable fibers; nanotubes may be oxidized or shortened. It follows that the risk assessment of the composites cannot be solely based on the properties of the fibers put into the composites, but on the properties of the fibers released from the composites, with the understanding that the properties before and after release are closely linked. This review is not intended to be an exhaustive review of the release studies. Instead, it focuses on the transformation of the fibrous fillers after their release from composites, especially the change of the properties essential for the health impacts.

## Transformation of the asbestos from construction materials

### Background

Asbestos is a family of six natural silicate minerals, containing long chains of silicon and oxygen that give rise to the fibrous nature of the mineral [15]. Asbestos is recognized as a carcinogen and it has been more than 30 years since the first national ban on asbestos in 1983 by Iceland [16]. To date, all the EU member states have banned usage of all forms of asbestos [17]. However, asbestos fibers still exist in a large number of products and the end-of-the-life treatment of asbestos-containing materials poses potential risks. An estimated 20% of buildings in the US still contain products such as shingles, cement pipes and insulation made from chrysotile asbestos [15]. Yet well-maintained asbestos in buildings will not spontaneously shed fibers into the air. Instead decay, renovation or demolition of the structures can lead to the release of fibers [15].

The most common methodology of asbestos waste management is the disposal in special landfills for toxic and hazardous wastes [18]. However, identifying an appropriate location for the installation of these landfills is difficult, due to the specific requirements of these sites according to current legislation and due to common operational difficulties [18].

Waste incineration is becoming a popular method to significantly reduce the volume of the deposited waste and to avoid soil contamination. For example, the current Swiss Technical Ordinance on Waste demands that all combustible waste has to be burned before deposition. Therefore landfilling of wastes containing asbestos and high fraction of organic contents are forbidden. Incineration of such wastes in municipal solid waste incineration (MSWI) plants and deposition of the slags and filter ashes afterward seem to be a solution, because it is known that thermal treatment could destroy the fibrous structure of the asbestos, transforming the asbestos to non-hazardous materials [19, 20].

Standard detection methods for asbestos fibers are usually based on filter collection and microscopic inspection. The National Institute for Occupational Safety and Health (NIOSH) has four standard methods for the analysis of asbestos fibers [21–24]. Two of them are for the analysis of filter samples by microscopy (phase contrast microscopy for method 7400, and transmission electron microscopy TEM for method 7402). The two other methods are for the analysis of powder samples, either by X-ray diffraction (method 9000) or by polarized light microscopy (method 9002). The EPA has also a standard method for the analysis of asbestos. Their procedure involves two mandatory steps of analysis by microscopy (a stereomicroscopic examination, followed by polarized light microscopy) for the qualitative classification of the fibers. The amount of asbestos in a residue can then be quantified by gravimetry, X-ray diffraction (XRD), polarized light microscopy, or analytical electron microscopy. There are also several other standard methods available e.g. [25, 26]. Many previous studies used electron microscopy and XRD to investigate the modification of the asbestos after thermal treatment.

### Transformation of asbestos by thermal treatment

**Fig. 1:**
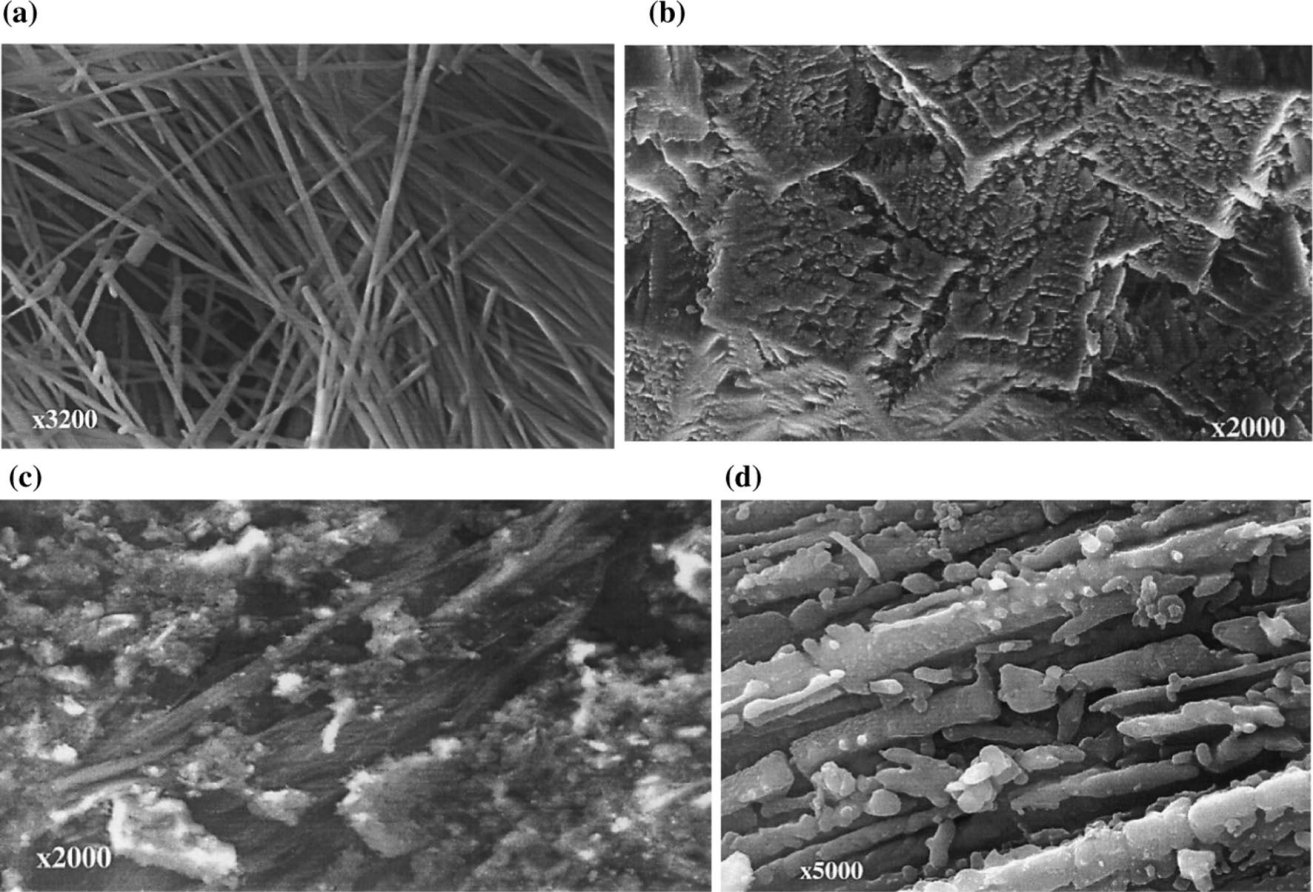
SEM images of **a** the initial sample of a pure amphibole asbestos; **b** the pure amphibole asbestos sample after thermal treatment; **c** the initial sample of a commercial asbestos-cement for external roofs pipes; **d** the asbestos-cement sample after thermal treatment. (Adapted from [20], with the permission of Elsevier)

Gualtieri and Tartaglia [20] reported that asbestos could be entirely transformed to a mixture of non-hazardous silicate phases throughout a thermal treatment at 1000–1250 °C and to a silicate glass at T > 1250 °C. They investigated four samples, including a pure serpentine asbestos, a pure amphibole asbestos, a commercial asbestos containing material utilized in the past for asbestos–cement pipes, and a commercial asbestos-cement for external roof pipes. Initially the pure asbestos samples had lengths over 10 μm and diameters less than 1 μm (Fig. 1a). After the thermal treatment, the asbestos samples lost the fibrous morphology and were transformed to non-hazardous silicate phases (Fig. 1b). The construction material samples had asbestos fibers dispersed in the heterogeneous matrix (Fig. 1c). The thermal treatment resulted in crystals of the silicate phases in place of the fibers (Fig. 1d). The authors also described the recycle of the thermally treated asbestos containing samples as a raw material for glass ceramics and traditional ceramics.

Gualtieri et al. [27] used time-resolved synchrotron powder diffraction to follow the thermal transformation of a cement-asbestos sample. The instrumentation allowed for the observation of metastable phases during the transformation of asbestos fibers into non-fibrous crystalline phases. The changing gas atmosphere in the closed system was shown to affect the final composition of the recrystallized product.

Gualtieri et al. [3] used environmental scanning electron microscopy to follow in situ the thermal transformation of chrysotile fibers present in cement-asbestos. It was found that the reaction kinetics of thermal transformation of chrysotile was highly slowed down in the presence of water vapor in the experimental chamber with respect to He. This was explained by chemisorbed water on the surface of the fibers which affected the dehydroxylation reaction and consequently the recrystallization into Mg-silicates.

Zaremba et al. [28] reported the possibility of detoxification of chrysotile asbestos through a low temperature heating and grinding treatment. They found that an isothermal treatment at 650 °C for at least 3 h caused the complete dehydroxylation of chrysotile Mg_3_Si_2_O_5_(OH)_4_. Transformation of the dehydroxylated phase to forsterite Mg_2_SiO_4_ was obtained by heat treatment in the range 650–725 °C. In addition, it was easily milled to pulverulent-shape material by mechanical milling.

Kusiorowski et al. [29] investigated thermal decomposition of 10 different samples of raw natural asbestos. They found that different temperatures were required (about 700–800 °C for chrysotile and more than 900 °C for amphibole asbestos). As a result of this process, the mineral structure was changed through dehydroxylation which led to the formation of X-ray amorphous and anhydrous phase. Kusiorowski et al. [30] extended their study to three asbestos–cement samples from different factories. Calcination of asbestos–cement wastes at ~1000 °C was sufficient to totally destroy the dangerous structure of asbestos. No significant differences in thermal decomposition among the types of asbestos–cement samples used were observed.

Yamamoto et al. [31] investigated simulated slag samples produced by high-temperature melting of asbestos-containing wastes. Fiber concentrations were below the quantification limit of their TEM-based method in all samples.

Transformation of the asbestos by the thermal treatment can be identified not only by microscopy, but also by other analytical techniques. Gualtieri et al. [27] used the synchrotron powder diffraction to observe the phase change of asbestos fibers. The Fourier transform infrared spectroscopy (FT-IR) spectra of asbestos normally show a characteristic double peak at 3640–3680/cm corresponding to the OH-stretching vibration. Upon thermal decomposition, the double peak disappeared [30]. Heating the samples causes appreciable other changes in their FT-IR spectra, which provides important information about the structural transformations. For instance, Kusiorowski et al. [29] showed that a characteristic triplet in the region 935–1080/cm, which is typical of the Si–O–Si stretches in the silica network, was clearly shifted toward lower frequencies.

### Discussion

The cited studies show that thermal treatment can be an effective solution to transform both raw asbestos samples and asbestos-containing construction materials into non-hazardous phase. Effective treatment of asbestos-containing cement wastes needs about 1000 °C. During the incineration process, asbestos may stay embedded or be liberated from the matrix and carried away by the air flow and thermal plume. Therefore, the asbestos may remain in the slag or become free standing. According to the directives of the European Union on the incineration of wastes, the gas resulting from the process must reach at least 850 °C. Moreover, if hazardous wastes contain more than 1% of halogenated organic substances, the temperature has to be raised to 1100 °C for at least 2 s during incineration [32]. Therefore, the temperature in the incineration processes may or may not be high enough for effective treatment of asbestos-containing wastes.

Currently there is no uniform practice in Switzerland regarding the incineration of wastes that contain asbestos in MSWIs and some MSWIs do accept small volumes [33]. In addition, the temperature is heterogeneous in an incinerator therefore asbestos fibers may have different degrees of thermal decomposition. The liberated asbestos may have long enough residence time in the incinerator to be thermally transformed; they may settle down as part of the slag or be carried by the flue gas and captured as part of the filter ashes. The distribution fractions are not known. Further studies are needed to investigate the fate and stability of asbestos fibers in MSWIs and to assess the risks for the operators and environment.

## Transformation of the released carbon fibers from composites

### Background

Carbon fibers are fibrous structures composed mostly of carbon atoms, which can be derived from organic fibers by subjecting them to high temperatures that drive off the non-carbon components [8]. Carbon fibers have been used in high performance applications from airplanes to automobiles and from satellites to sporting goods [34]. Carbonized fibers include carbon (amorphous) and graphite (crystalline; made by further heating amorphous carbon fibers) [8]. All commercial carbon fibers produced today are based on rayon (a cellulose-based polymer), PAN (polyacrylonitrile fiber) or pitch (a tar-like mixture of hundreds of branched organic compounds) [34]. PAN-based fibers have superior tensile strength; pitch-based fibers are unique in their ability to achieve ultrahigh Young’s modulus and thermal conductivity [34].

Carbon fiber reinforced polymer (CFRP) composites have gained great attention due to their interesting combination of strength, durability, high strength-to-weight ratios and corrosion resistance [35]. They are finding increasing applications in architecture, aerospace, automotive, and sporting goods industry [35, 36]. Release of carbon fibers from CFRPs has been observed during machining [37–40] and during tensile strength tests [41]. The fiber content of CFRPs is often above 50 vol% which means that the produced dust during machining or tensile tests consists mainly of materials from the fibers. Besides fibers with the same diameter as the embedded fibers in the composite, respirable fibers with smaller diameters were also generated, indicating transformation of the embedded fibers during the process.

Previous studies showed that the toxicity of carbon fibers depended on their sizes. Holt and Horne [39] exposed guinea pigs to dust obtained by feeding PAN-based carbon fibers into a hammer mill. The nonfibrous particles in the dust were phagocytosed. The few carbon fibers found in the lung that were longer than 5 μm were still extracellular after 27 weeks and they were uncoated. No pathological effects were observed. Warheit et al. [42] exposed rats to PAN-based carbon fibers which were 9 μm in diameter and considered to be non-respirable. They had no effect on any of the parameters tested. In the same study, the pitch-based carbon fibers with <2 μm aerodynamic diameter produced a dose-dependent transient inflammatory response in the lungs of exposed rats. Martin et al. [43] investigated the cytotoxicity of particles generated during machining of CFRP composites (characterized by [38]. For two samples they saw a slight toxicity, but as the particles consisted of fibers and matrix materials, it was not clear what caused the effect.

### Carbon fiber release from composites and transformation

Holt and Horne [39] fed PAN-based carbon fibers into a hammer mill and examined the dust taken from the air in the dusting chamber. They observed fibers about 10 μm in diameter and >100 μm long, which might have the same diameter as the original ones. Only low concentrations of dust of respirable size were produced and less than 1% of the respirable carbon particles were fibrous. The respirable black fibers had diameters about 1–2.5 μm and lengths up to 15 μm. They were possibly fragmented fibers from the original ones, though the authors did not explain where these smaller fibers were from. It appeared that the authors used an optical microscope therefore the size resolution was limited. The authors used the generated dust for toxicity tests in guinea pigs and found no adverse effects.

Henry et al. [44] analyzed airborne dust during preparation and machining of carbon fiber composites at a PAN-based production facility and reported 0.01–0.0002 f/ml (mean diameters >6 μm and mean lengths >30 μm). Gieske et al. [45] reported concentrations of 0.001–0.05 f/ml (mean diameters >5.5 μm and mean lengths >900 μm) during various phases of carbon fiber production. Based on the data they reviewed, Warheit et al. [8] suggested that released carbon fibers tended to be non-respirable—diameters were 3.9–7.8 μm and lengths were 32.8–2342 μm.

Mazumder et al. [40] investigated aerodynamic and morphological properties of the fibers and fiber fragments released from commercial laminates containing carbon and graphite fibers during cutting, grinding and by thermal degradation. The virgin fiber diameters were 5.8–8.0 μm and fiber volume content was about 60%. The authors found that mechanical chopping of virgin carbon fibers produced sharp-edged fiber like particles in the respirable size range. When the composites were subjected to grinding, fibers were often exposed from their polymer matrix, and the released particles contained small fibers and fragments with irregular shapes, and a significant number of fibers having smaller diameters than the original ones and sharp edges because of fibrillation. The authors showed electron micrographs demonstrating how the fiber could split, generating particles with irregular shapes and often with sharp edges. It was estimated that about 90% did not have such sharp edges. They concluded that fiber fragments in the sub-micrometer range could be generated during the machining process.

Mazumder et al. [40] exposed virgin carbon fibers to a temperature of 850 °C for 4.5 h. They observed that the fibers underwent significant fragmentation during oxidation, and apparently lost their crystalline property. Debris in this process from carbon fibers primarily consisted of amorphous carbon particles rather than fiber like particles. When the particles released from grinding of the composites were heated, the epoxy resin evaporated quickly at temperatures above 400 °C, then the vapor condensed to form respirable particles.

Boatman et al. [38] performed machining operations on six carbon fiber/epoxy composites and analyzed the released dust. By microscopy, bulk particles ranged from 7 to 11 μm in diameter, with mean aspect ratios from 4 to 8:1. The relative fractions of respirable to total mass of bulk samples were <3%. The authors concluded that under their machining protocols, dusts at the tool face contained few particles of respirable size with no evidence of splitting of fibers longitudinally.

Bello et al. [37] investigated release of airborne particles during dry and wet cutting of composites containing CNTs and carbon fibers. The carbon fibers had nominal diameter of 6–7 μm and broken fibers of about the same diameter were observed in the dust. Fibers which might have originated from fracturing of the carbon fibers along their axis were identified, which were typically thinner, longer, and had higher aspect ratios than those associated with the broken carbon fibers. Bello et al. [46] performed drilling on the same composites and again found both carbon fibers fractured perpendicular to the fiber axis and split fragments along the axis.

Schlagenhauf et al. [41] investigated the fiber release and possible risk for the operating staff in two CFRP cable tensile tests. The carbon fibers had a filament diameter of 5 μm and were pre-impregnated by an epoxy polymer resin matrix. The fiber volume fraction was 60% in the cables. The tensile tests involved first loading the cables with very high elastic energy, then releasing the energy abruptly in the failure event, which caused the cable to rupture and induced a vast amount of dust. Measurements with aerosol devices and examination of the filter samples showed that the cable failure caused release of particles and free-standing fibers whereof a fraction had diameters below 3 μm and thus were respirable according to WHO [12]. The measured peak fiber concentration of 0.76 fibers/cm^3^ and calculated concentration of 0.07 fibers/cm^3^ for an 8 h time weighed average were below the PEL of 1 respirable fiber/cm^3^ given by OSHA. The peak fiber concentration was close to the PEL indicating needs for protective measures for the workers during and immediately after the tensile tests.

**Fig. 2:**
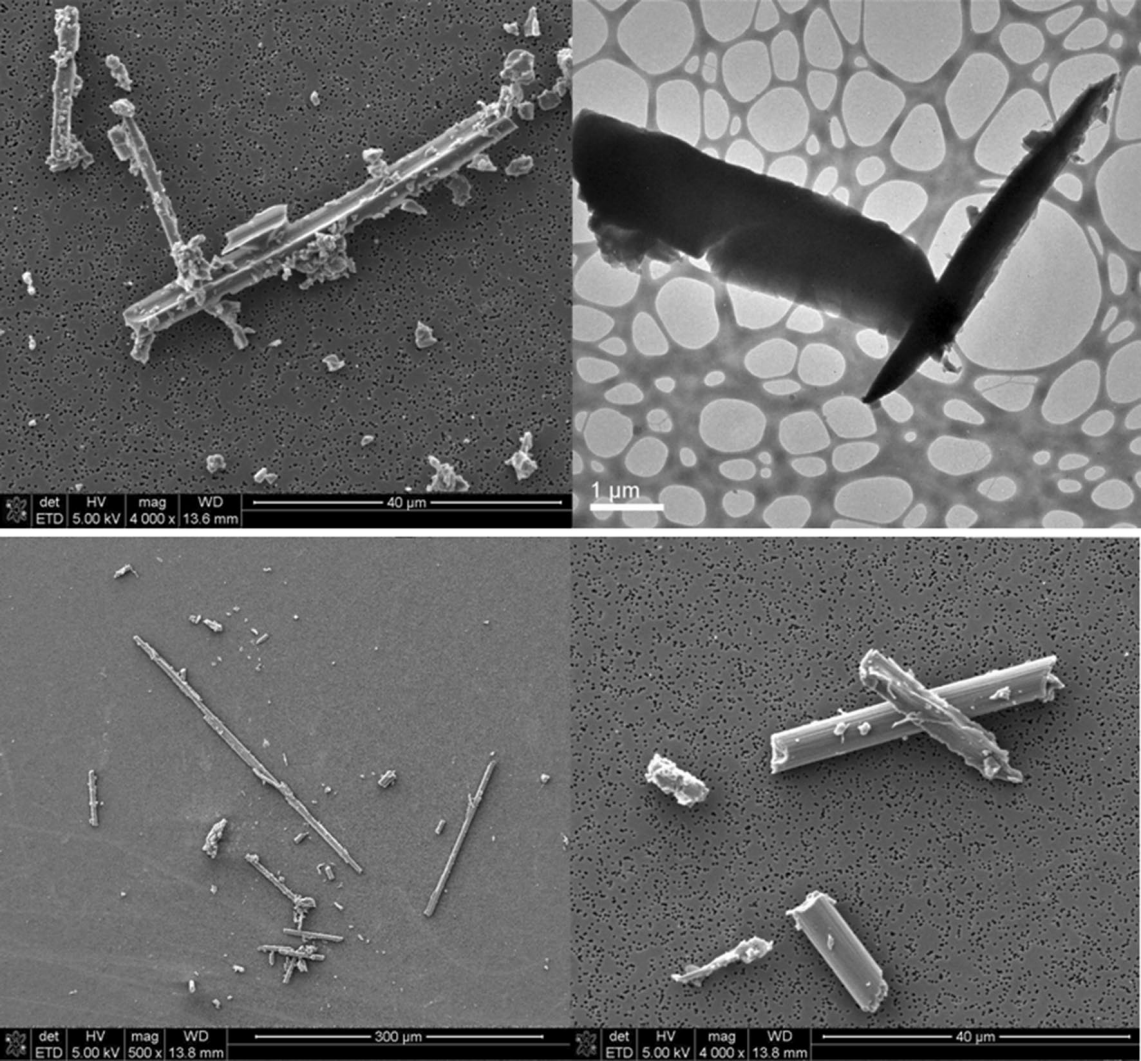
Example SEM and TEM images of the released particles following the rupture of CFRP cables in the tensile strength test. (Partially adapted from [41]

Example SEM and TEM images from the study of Schlagenhauf et al. [41] are shown in Fig. 2. Some collected fibers had the original diameter of 5 µm and relatively smooth surface decorated by residual particles. These fibers were not considered to be respirable according to the WHO criteria. There were also more fibers which appeared to be split during the cable failure and could be respirable due to the diameters below 3 µm. Further, fragmented particles from the composite matrix and fibers, whereof most had diameters below 10 µm, were also collected. The TEM images show a few fibers with a diameter below 1 µm. The morphologies of the released fibers suggested that the embedded fibers were not only severed perpendicular to the fiber axis, but also along the fiber axis in many instances, causing respirable fibers with smaller diameters.

### Discussion

The studies reviewed here demonstrate that the carbon fibers with diameters 5–10 µm embedded in composites can be released by mechanical operations. The released fibers may be broken perpendicular to the fiber axis, thus with the same diameters as the original ones; they may also be fractured or split along the fiber axis, leading to respirable fibers with smaller diameters. This splitting can be explained by the micro structure of the fibers. Diefendorf and Tokarsky [47] described carbon fibers as composed of a ladder structure of graphite layers that are aligned parallel to the fiber axis. Endo and Dresselhaus [48] described several different carbon fiber structures. The PAN-based fibers consist of small carbon structural units preferentially aligned with the carbon hexagonal segments parallel to the fiber axis, and the intertwined morphology is responsible for the high mechanical strength of the PAN-based fibers. The mesophase pitch-based fibers consist of well aligned graphitic layers nearly parallel to the fiber axis, and this high degree of crystallinity is responsible for their high modulus or stiffness. The vapor grown fibers consist of coaxial cylindrical graphene sheets and are closely related to multiwall carbon nanotubes. The graphite whiskers [49] were reported to have the scroll structure of rolled up graphite sheets. The bonds between the graphitic layers or sheets or small carbon structural units are relatively weak and susceptible to fragmentation. The fibers used by Schlagenhauf et al. [41] were PAN-based (Tenax^®^ IMS 60, Teijin, Tokyo, Japan) and the split fiber in the first panel of Fig. 2 showing that a small carbon structural unit broke off from the large fiber. Mazumder et al. [40] described some of their observed fragmented fibers with shattered outer graphite structure, exposing the inner core of the fiber. These fibers seemed to have the scroll structure of rolled up graphite sheets.

Thermal treatment can cause both asbestos and carbon fibers to lose their fibrous structures. However, the mechanisms are different. The asbestos is subjected to the dehydroxylation reaction and consequently the recrystallization into the silicate phase. The carbon fibers may undergo fragmentation during oxidation, lose their crystalline property and turn into amorphous carbon particles.

## Transformation of the released carbon nanotubes

### Background

CNTs represent a type of fascinating nanomaterial which gained numerous applications due to their special mechanical, electrical, thermal and optical properties [4, 50, 51]. As filler in composites, CNTs can lead to superior or additional properties compared to their neat matrix materials including tensile strength and Young’s modulus [52], energy absorption [53], improved scratch and wear resistance [54], electrical and thermal conductivity [55, 56], fire resistance [57], and optical properties [58].

CNTs are also one of the most heavily studied nanomaterials for their potential impacts on human and environment [59–67] among the others). Therefore, voluminous studies have been dedicated to the release of CNTs from composites [13, 14, 37, 46, 68–88]. The release of CNTs by mechanical stresses and weathering or a combination of them has been widely investigated.

Another possible release route is by thermal treatment which decomposes the polymer matrix and exposes the CNTs. CNTs possess high thermal stability. Pang et al. [89] studied the oxidation of CNTs by thermogravimetric analysis (TGA) in air. The maximum rate of weight loss took place at 695 °C at a heating rate of 1 °C/min. The oxidative stability of CNTs is dependent on the defects and tube diameter [90]. The defects are present at the ends, bends, Y-junctions, and kinks in nanotubes and they contribute to a decrease in the oxidative stability. A smaller diameter results in a higher degree of curvature and subsequently a higher reactivity toward oxygen. Bom et al. [90] showed that thermal annealing could remove the defects and improve the thermal stability of the multiple wall carbon nanotubes (MWCNTs). By annealing at 2800 °C, Bom et al. showed the oxidative stability enhancements of MWCNTs was 155 °C, and complete decomposition of the annealed MWCNTs needed temperatures around 800 °C. CNTs are considered to be a promising flame retardant to replace the conventional halogenated ones [57]. The CNT nanocomposites may be exposed to high temperatures in a fire accident, in incineration plants, or in a thermal treatment intended to recover the CNTs for reuse. The scenarios will be discussed.

### Transformation of the released CNTs from composites

In the study of Bello et al. [37] composites containing CNTs and carbon fibers were subjected to dry and wet cutting. Although release of chopped and split carbon fibers was reported, no released CNTs were detected. In a subsequent study, Bello et al. [46] performed drilling on the composites and observed release of clusters of CNT aggregates. In both of the above studies, the authors observed submicron fibers with at least one nanoscale dimension without discussing their origins. The CNTs in the carbon-fiber based composites were reported to be 8 nm in average diameter and 100–150 µm long [37]. The released CNT aggregates had complex morphology and the diameter and length of the involved CNTs were not reported.

Cena and Peters [69] reported that weighing bulk CNTs and sanding epoxy containing CNTs generated few airborne nano-sized particles. Sanding epoxy containing CNTs might generate micrometer-sized particles with CNTs protruding from the main particle core. The protruding CNTs had diameters (~25 nm) in the range of the original CNTs (10–50 nm). No free standing CNTs were found. Huang et al. [74] reported more results for sanding epoxy sticks with CNTs. Similar results were obtained in that protruding CNTs with diameters around 25 nm were observed. The authors did not detect rod shaped particles from micrographs, except for the tests conducted with 4% CNT epoxy, in which particles with features consistent with free CNTs were observed.

**Fig. 3:**
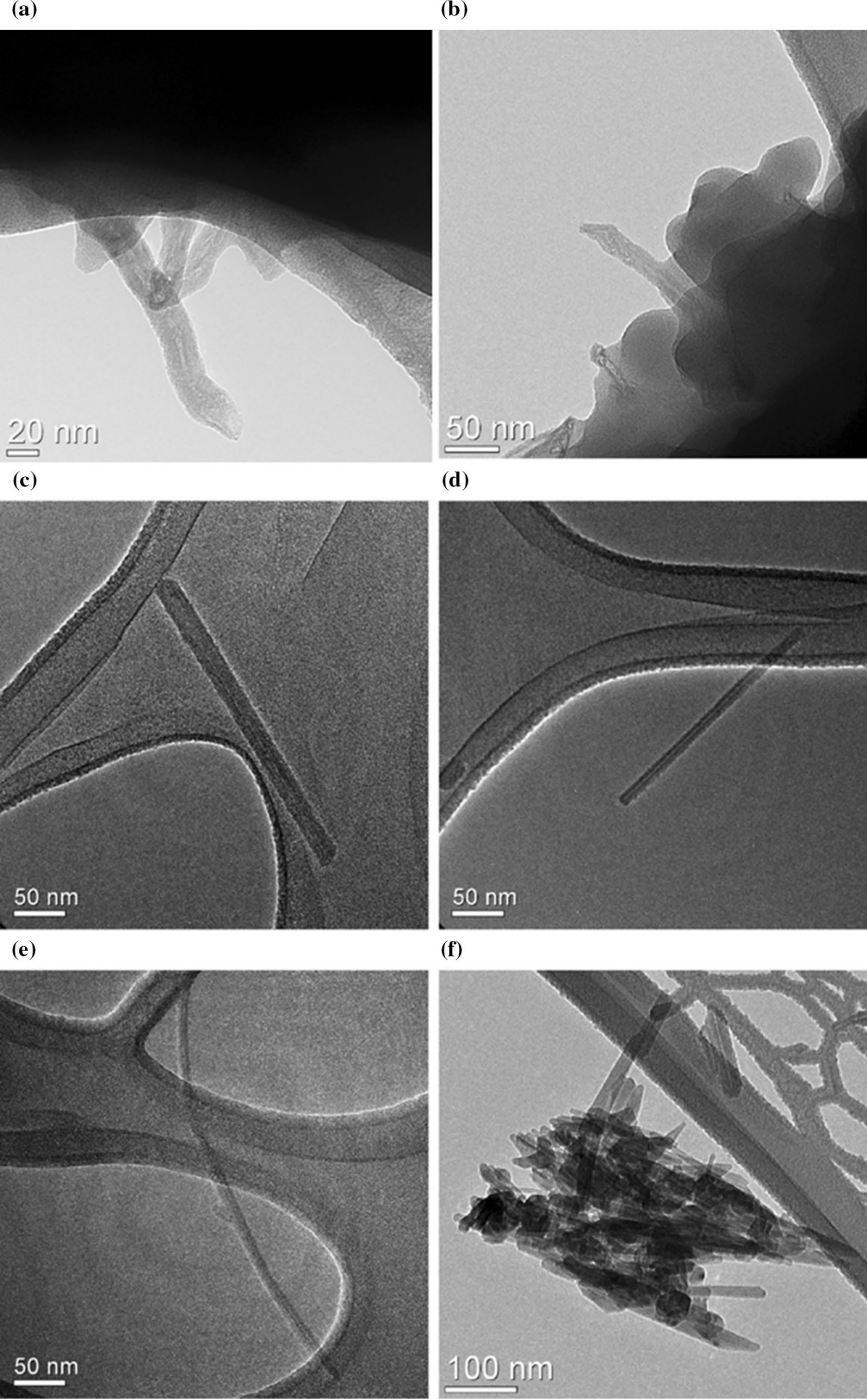
TEM images of abraded particles from a CNT/Epoxy composite by the Taber Abraser. **a**, **b** Protruding CNTs from abraded particles of the 1 w% CNT composite; **c**–**e** free-standing individual CNTs; **f** an agglomerate of CNTs with a couple of individual CNTs scattered nearby. (Partially adapted from [80]

Schlagenhauf et al. [80] used the Taber Abraser to perform abrasion on a CNT/epoxy composite, for which the properties were reported in Hollertz et al. [55]. MWCNTs (Baytubes, C150p) with 1–10 µm lengths and 13–16 nm outer mean diameters were used to produce the composites. The MWCNT mass content was 0.1 or 1% and they were dispersed in the epoxy resin by three-roll milling at a gap pressure of 1 MPa. The composite preparation process evidently reduced the CNT lengths to about 0.7 ± 0.2 μm [55]. After abrasion, protruding CNTs from the released epoxy particles were visible (Fig. 3a, b), and a non-negligible amount of free-standing CNTs (Fig. 3c–e) and agglomerates of CNTs were also found (Fig. 3f). The released CNTs had about the same diameters as the original ones. However, the average length of 19 imaged free-standing CNTs was 304 ± 251 nm. Therefore the released CNTs were shortened during the abrasion process compared to the embedded ones. Schlagenhauf et al. [81] developed an ion labeling method to quantify the exposed CNTs in the respirable fraction of the abraded particles, and found approximately 4000 ppm of the MWCNTs were released as protruding or free-standing MWCNTs (which could contact lung cells upon inhalation) and approximately 40 ppm as free-standing MWCNTs in the worst-case scenario.

Golanski et al. [71] performed abrasion on polycarbonate, epoxy and PA (polyamide) polymer composites containing CNTs up to 4 wt%. They developed practical tools inducing non-standardized high stresses such as mechanical shocks and hard scratches simulated by a metallic brush. No release of CNTs was measured for the samples with well dispersed CNTs, however for the samples with poorly distributed CNTs, individual free standing CNTs were observed on TEM grids. The CNTs used in the study had an external diameter of 12 nm. The authors did not give size information for the released free standing CNTs.

Ogura et al. [91] investigated the particle release caused by the grinding of polystyrene-based composites containing 5 wt% single-wall carbon nanotubes (SWCNTs). Free-standing CNTs were not observed, whereas micron-sized particles with protruding fibers speculated to be CNTs were observed. The CNTs had a tube diameter of approximately 3 nm and it is difficult to confirm the fibers were CNTs from the SEM images of the released particles.

Nguyen et al. [77] and Petersen et al. [78] investigated the degradation of a CNT/epoxy nanocomposite under intensive UV-light. UV-light can cause oxidation of the polymer and chain scission therefore damage of the sample surface. The studies showed that the epoxy-rich surface layer of the nanocomposite was removed relatively rapidly, leaving a surface covered almost completely with a network of MWCNTs. The MWCNT network on the weathered epoxy surface was more mechanically resistant to scratching than the neat epoxy. The authors’ analysis of released particles did not show free standing CNTs. The strong mechanical properties of the CNT network and the lack of broken CNTs implied that the UV exposure did not damage the integrity of the CNTs. Ging et al. [92] evaluated the degradation of a CNT/epoxy nanocomposite with neat and amino functionalized CNTs exposed to the combination of UV, moisture, mechanical stress and other factors. Several possible forms of CNTs were found on the composite surface by UV irradiation: completely unprotected and agglomerated CNTs; partially exposed CNTs fractured due to the crack formation originating from exposure; CNTs still encapsulated in the matrix; and fragments of the matrix.

Wohlleben et al. [87, 88] analyzed degradation scenarios for different nanocomposite materials. They found after long term weathering the polymer matrix (polyoxymethlene POM and thermoplastic polyurethane TPU) with embedded CNTs degraded and exposed the nanofiller as an entangled CNT network. Immersion in water did not lead to release of CNTs from the network. Hirth et al. [73] investigated sanding and weathering of CNT/epoxy nanocomposites and observed embedded or protruding CNTs in the released particles. The authors identified the protrusions from mechanically released fragments unambiguously as naked CNTs by chemically resolved microscopy. The protruding CNTs matched the morphology and diameter of original CNTs and formed a surface layer with length around 0.3 µm. The original CNTs had 10–50 nm outer diameter and 1–20 µm length. In the weathering experiments, protruding networks of CNTs remained after photochemical degradation of the matrix, and it took the worst case combinations of weathering plus high-shear wear to release free CNTs in the order of mg/m^2^/year.

Schlagenhauf et al. [82] performed weathering studies on a CNT/epoxy nanocomposite by both UV exposure and immersion in water. In the UV exposure experiments, the authors did not observe the accumulated CNT layer on the degraded surface as in Nguyen et al. [77], Petersen et al. [78] and Wohlleben et al. [87, 88]. Instead, the results indicated that delamination occurred between the exposure times of 1000–1500 h and the top layer of the surface fell off the composite. The remaining surface was relatively smooth with low degrees of chemical degradation. The difference with other weathering studies might be due to the much larger thickness of the samples and lower relative humidity in Schlagenhauf et al. [82].

Kashiwagi et al. [57, 93, 94] performed a series of experiments to investigate the thermal degradation and flammability properties of CNT composites. Kashiwagi et al. [57] showed that MWCNTs enhanced the thermal stability of polypropylene (PP). They concluded that the flame retardant performance was achieved through the formation of a relatively uniform network-structured floccule layer covering the entire sample surface. This layer re-emitted much of the incident radiation back into the gas phase from its hot surface and thus reduced the transmitted flux to the receding PP layers below it, slowing the PP pyrolysis rate. This network-structured layer was formed during cone calorimeter experiments below around 600 °C. Kashiwagi et al. [57] described the network-structured layer as partially oxidized CNTs embedded in an agglomerate composed of iron oxide primary particles. The iron was the catalyst for the used CNTs. Kashiwagi et al. [94] stated that the tubes in the network were more ‘intertwined’ and larger than those in the original sample. The tubes were also partially oxidized. The mass of the network layer was very close to the initial mass of carbon nanotubes in the original nanocomposite. Kashiwagi et al. [93] extended their studies to poly(methylmethacrylate) (PMMA), SWCNTs and carbon nanofibers and obtained similar results.

The formation of CNT network in the combustion residuals was confirmed in a number of studies of fire behaviors and flame retardants [95–99]. These studies covered different types of composite matrices such as polyamide 6 (PA6), silicone foams, polyethylene naphthalate (PEN), PP/wood flour and nanofillers including pristine CNTs, hydroxylated CNTs, carbon black, graphite and graphene.

In the recent years, several studies focusing on CNT release during thermal treatment of nanocomposites have been published. Bouillard et al. [68] used an acrylonitrile butadiene styrene (ABS) composite with 3 wt% of CNTs, combusted the sample in a furnace and collected released particles on TEM grids. The MWCNTs used for composite production had the mean outer diameter of 10–15 nm and length of 0.1–15 μm. They found that MWCNTs of about 12-nm diameter and 600-nm length were released to the air during combustion; these dimensions were very similar to those of the original MWCNTs. The released numbers were quite significant posing a possible sanitary risk in the case of accidental scenarios. The authors observed several isolated (not in bundle) CNT fibers, as well as CNT fibers in bundles. These CNTs had the catalysts remaining attached to their ends and had shapes and chemical speciation similar to those of the original MWCNTs.

Schlagenhauf et al. [83] thermally decomposed MWCNT/epoxy composites in a tube furnace under air or nitrogen atmosphere. The temperature was gradually increased and a large number of airborne particles were released at temperatures below 300 °C when air was used. The usage of a thermal denuder showed that only 0.01 wt% of the released mass consisted of non-volatile particles. A release of free-standing MWCNTs was not observed.

Sotiriou et al. [85, 86] setup a system to decompose composites in tube furnaces and characterize the released aerosols. In the experiments of a polyurethane (PU)/MWCNT composite at 500 and 800 °C, they found no CNTs in the released aerosols. Residual ash existed only at 500 °C and numerous CNT protrusions were observed from the surface of the ash. The authors inferred that the CNTs were intact because 500 °C was below the expected oxidization temperature for MWCNTs. Singh et al. [84] extended the experiments to CNT composites with polypropylene and polycarbonate matrices and found no CNTs in the released aerosols as in previous studies.

Vilar et al. [100] investigated calcination as a method to recover nanomaterials from nanocomposites. They used PA6 composites containing pristine MWCNTs or MWCNTs modified to be more compatible with PA6. The calcination conditions were 410 °C during 3 h 30 min. The temperature was set with the consideration that the polymer was burned and CNTs did not suffer any change. Calcinated nanomaterials were characterized by FT-IR and TGA and neither of the two analyses showed any difference between the nanomaterials before and after calcinations. However, electron microscopy showed that the MWCNTs recovered from composites were with a small amount of attached polymer.

### Discussion

The studies on release of CNTs from nanocomposites mostly focused on whether the CNTs were released; information on the transformation of the physical and chemical properties of the released CNTs is sparse. From the analytical point of view, it is difficult to obtain accurate measurement of the properties of released CNTs when they are scattered or embedded in fragments of the matrix, therefore to prove the change of properties. The number of studies showing release of free standing CNTs without any matrix material is very limited, and amount of the free CNTs usually did not allow sophisticated analysis.

The toxicity of the released CNTs together with the fragmented matrix particles can be different from the pristine CNTs, therefore, toxicity tests are important to understand the health impact of the particles released from composites, especially in the cases where CNT release is detected. These tests are different from the mechanistic toxicity study of the pristine material, as they are designed to answer practical questions related to real world applications. The toxicity of the released particles by mechanical abrasion from CNT-nanocomposites has been investigated by several in vitro and in vivo studies. Wohlleben et al. [87, 88], Ging et al. [92] and Saber et al. [101] did not detect release of the CNTs and found no additional toxic effect caused by the added nanofillers in comparison with the neat matrix materials. Schlagenhauf et al. [81, 82] observed protruding CNTs from matrix particles and some free standing CNTs, however, the toxicity tests revealed that the abraded particles did not induce any acute cytotoxic effects. Ging et al. [92] and Schlagenhauf et al. [81, 82] all observed toxic effects of the virgin CNTs, but their no-effect observation from the released particles demonstrated that the health impact assessment needs to take the transformation during the release into account.

CNTs play distinct roles in the different scenarios where they are exposed to high temperatures. In the applications using CNTs as flame retardants, the CNTs are expected to form a protective network and impede the fire. In incineration plants, the ideal outcome is to decompose CNTs [14, 102] and to avoid exposure of CNTs to human. In recovery operations, the temperature needs to be high enough to burn off the polymer matrix but below the point where the CNTs are oxidized. The temperature dependence of the CNT’s oxidative stability is obviously critical. The temperature in an accidental fire is not controllable. Using the more thermally stable CNTs in the retardant applications befits the “safe by design” concept. The temperatures in incineration and recovery operations should be set according to the goals. Different types of CNTs have different diameters and defects, thus variable thermal properties. The fact that the CNTs are embedded in the polymer matrix and interaction of CNTs with the molten matrix further complicate the situation. In case the CNTs and their agglomerates are liberated from the matrix, their mobility and transportation may be complex in the flue gas and thermal plume [103]. More studies for the thermal behavior of different types of CNT composites are needed to address the topic. Harper et al. [14] considered the release of CNTs from waste incineration to be low given CNTs can be combusted; even if the CNTs survive the incineration, they may end up in bottom ash or fly ash captured by the filters, and eventually in the landfill.

## Summary

We reviewed studies on release of fibrous fillers in composites and identified a number of scenarios where the physical and chemical properties of the released fibers may be altered. A summary of the possible transformation of the released fibrous fillers is shown in Table 1.

**Table 1:** Summary of the possible transformation of the released fibrous fillers from composites

Fibrous filler	Composite matrix	Release process	Transformation of released fibers	References
Asbestos	Cement; other construction materials	Thermal treatment of about 1000 °C and above	Asbestos were transformed to non-hazardous silicate phase	Gualtieri and Tartaglia [20], Gualtieri et al. [3, 27], Kusiorowski et al [30], Yamamoto et al. [31]
Different carbon fibers	Epoxy	Hammer mill, dry and wet cutting, grinding, drilling	Fibers split along the axis from the original fibers were released. They had smaller diameters and might be respirable	Holt and Horne [39], Mazumder et al. [40], Bello et al. [37, 46]
Different carbon fibers	Epoxy	Heating to 400 and 850 °C	At 850 °C, fibers underwent fragmentation during oxidation, and lost crystalline property	Mazumder et al. [40]
PAN-based carbon fibers	Polymer cable	Tensile stress test to cable failure	Respirable fibers split along the fiber axis from the original fibers were released	Schlagenhauf et al. [41]
CNTs	Epoxy	Sanding	CNTs protruding from fragments of matrix material had similar diameters as the original ones.	Cena and Peters [69], Huang et al. [56]
CNTs	Epoxy	Abrasion	Free standing single and agglomerated CNTs were released and had average length (304 nm) shorter than the CNTs in the matrix (0.7 µm)	Schlagenhauf et al. [80]
CNTs	Epoxy; POM; TPU	UV exposure	Surface of the sample was covered by a network of CNTs and their integrity was not damaged	Nguyen et al. [77], Petersen et al. [78], Wohlleben et al. [87, 88]
CNTs	Epoxy	Combination of sanding and weathering	Protruding CNTs had the same diameter as the original ones and formed a surface layer with length around 0.3 µm, shorter than the original length of 1–20 µm	Hirth et al. [73]
CNTs; carbon nanofibers	PP; PMMA	Fire test	A protective CNT network was formed in the combustion residuals. The tubes in the network were more ‘intertwined’ and larger than the original ones. The tubes were partially oxidized. Iron catalysts were also oxidized	Kashiwagi et al. [57, 93, 94]
CNTs	ABS	Combustion	Free isolated and bundled CNTs were released to the air with dimensions similar to the original MWCNTs	Bouillard et al. [68]
CNTs	PU	Thermal decomposition	CNT protrusions were observed from the surface of the ash and CNTs were assumed to be intact	Sotiriou et al. [85, 86]
Pristine and compatibilized CNTs	PA6	Calcination	Recovered CNTs showed no difference from the original ones by FT-IR or TGA analysis but showed a small amount of attached polymer in TEM	Vilar et al. [100]

*ABS* acrylonitrile butadiene styrene, *FT-IR* Fourier transform infrared spectroscopy, *PA6* polyamide 6, *PMMA* poly(methylmethacrylate), *POM* polyoxymethlene, *PP* polypropylene, *PU* polyurethane, *TGA* thermal gravimetric analysis, *TPU* thermoplastic polyurethane

The most important release scenario for asbestos now is the end-of-the-life treatment of asbestos-containing materials. A number of studies showed that thermal treatment transforms both raw asbestos samples and asbestos-containing construction materials into non-hazardous phase when the temperature was above about 1000 °C. The stability and fate of asbestos in real incineration operations with heterogeneous temperature and airflow still need further investigation.

Carbon fibers usually possess diameters in the range of 5–10 µm and are not considered respirable. However, mechanical operations on their composites can cause release of not only fibers with the same diameter as the embedded fibers, but also smaller respirable fibers caused by splitting of fibers along the axis. For nanomaterials, the weight percentage in composites lies in the low single digit range causing no or only a low number of released fibers when machined. However, carbon fibers can make up more than 50% of the volume of composites, therefore the released amount of fibrous particles can be substantial. The health impact could be concerning if the split carbon fibers with respirable sizes are present.

For CNT composites exposed to mechanical forces, Schlagenhauf et al. [13] concluded that the expected release scenarios include free standing CNTs, agglomerated CNTs, and particles with- and without protruding CNTs. Due to the nature of the release caused by mechanical forces, the released CNTs are possibly on average shorter than the CNTs in the composite. In all the reviewed studies, the diameters of the released CNTs were in the same range as the original CNTs used in the composites. Normally during the release processes, the CNT concentration in air is too low for them to agglomerate. This means that the finding of released CNT agglomerates can indicate a poor distribution of the CNTs in the investigated nanocomposite. The possible shorter length of the released CNTs indicates less toxicity to pulmonary cells and generally less persistent in the lung [8, 63]. The release process probably does not increase the agglomeration degree of the CNTs, therefore possibly not causing increased toxicity associated with agglomerates [104, 105]. The toxicity studies so far found no additional toxic effect caused by the added CNTs in comparison with the neat matrix materials; the observation may be mainly due to the low amount of released CNTs.

UV exposure can degrade the matrix and expose a layer of CNTs on the sample surface. Fire or thermal treatment can decompose the matrix and leave a network of CNTs in the residuals. Previous studies indicated that these CNT network structures had good mechanical strength and the CNT integrity was generally not damaged. CNTs were not easily liberated from these intertwined network structures. However, combined stresses such as weathering followed by additional shaking, abrasion, runoff water, etc., may cause CNTs release [14]. CNTs released by thermal treatment may be partially oxidized. Some studies showed that the CNTs oxidized by strong acid were more toxic than pristine CNTs [106, 107]. More studies on the thermally oxidized CNTs are needed.

The transformation scenarios of the released fibers from composites lead to different changes of the potential health impacts of the fibers. Thermal treatment can destroy the fibrous structure of asbestos and transform asbestos into non-hazardous phase. Mechanical operations and heating at certain temperatures may cause release of carbon fibers split along the fiber axis. The smaller diameters increase the deposition probability of the split carbon fibers in the gas exchange regions of the lung. The dose of such fibers may be high in occupational settings given the high volume fraction of carbon fibers in composites. Therefore the health impact of the embedded carbon fibers is probably increased following the transformation in the release process. A number of studies on the CNT release by mechanical operations, weathering and thermal treatment demonstrated that the released CNTs had similar diameters as the original ones, and the fiber integrity was largely undamaged. On the other hand, the released CNTs were on average shorter than the CNTs in the composite, therefore they would be easier to be cleared by the macrophage cells and less biopersistent. In summary, the released CNTs are possibly less harmful than the virgin CNTs.
